# Cystic Fibrosis Transmembrane Conductance Regulator (CFTR): CLOSED AND OPEN STATE CHANNEL MODELS*[Fn FN2]

**DOI:** 10.1074/jbc.M115.665125

**Published:** 2015-07-30

**Authors:** Valentina Corradi, Paola Vergani, D. Peter Tieleman

**Affiliations:** From the ‡ Department of Biological Sciences and Centre for Molecular Simulation, University of Calgary, Calgary, Alberta T2N 1N4, Canada and; § Research Department of Neuroscience, Physiology and Pharmacology, University College London, Gower Street, London WC1E 6BT, United Kingdom

**Keywords:** ABC transporter, chloride transport, cystic fibrosis, cystic fibrosis transmembrane conductance regulator (CFTR), molecular modeling

## Abstract

The cystic fibrosis transmembrane conductance regulator (CFTR) is a member of the ATP-binding cassette (ABC) transporter superfamily. CFTR controls the flow of anions through the apical membrane of epithelia. Dysfunctional CFTR causes the common lethal genetic disease cystic fibrosis. Transitions between open and closed states of CFTR are regulated by ATP binding and hydrolysis on the cytosolic nucleotide binding domains, which are coupled with the transmembrane (TM) domains forming the pathway for anion permeation. Lack of structural data hampers a global understanding of CFTR and thus the development of “rational” approaches directly targeting defective CFTR. In this work, we explored possible conformational states of the CFTR gating cycle by means of homology modeling. As templates, we used structures of homologous ABC transporters, namely TM(287–288), ABC-B10, McjD, and Sav1866. In the light of published experimental results, structural analysis of the transmembrane cavity suggests that the TM(287–288)-based CFTR model could correspond to a commonly occupied closed state, whereas the McjD-based model could represent an open state. The models capture the important role played by Phe-337 as a filter/gating residue and provide structural information on the conformational transition from closed to open channel.

## Teaser

**Background:** CFTR, which controls Cl^−^ permeation across epithelia, is an ABC transporter whose structure is not known.

**Results:** We modeled CFTR using the crystal structure of four homologous transporters.

**Conclusion:** We identified possible open and closed conformations of CFTR.

**Significance:** The models help the interpretation of experimental data and provide new hypotheses to test with experiments.

## Introduction

Cystic fibrosis is a monogenic disorder caused by mutations in the gene encoding the cystic fibrosis transmembrane conductance regulator (CFTR)^3^ (1), an anion channel present in several epithelia. Altered epithelial permeability leads to progressive organ deterioration and premature death. The deletion of Phe-508 in CFTR is responsible for the majority of cystic fibrosis cases and causes CFTR misfolding and retention in the endoplasmic reticulum (2), as well as defective channel function (3). However, more than 1000 mutations are linked to cystic fibrosis and interfere with its maturation, folding, or function (4–6). Defective CFTR function has also been implicated in the pathogenesis of several other disease processes, including chronic obstructive pulmonary disease, cholera, cardiogenic edema, and ovarian cancer (7–10). Since its discovery in 1989 (1, 11), major advances have been made in understanding how CFTR works, but the lack of crystallographic structures of the full-length CFTR still hampers a detailed understanding of its molecular mechanism.

CFTR belongs to the ATP-binding cassette (ABC) transporter superfamily (11), a class of membrane proteins that couple the hydrolysis of ATP with the transport of molecules across membranes (12, 13). Human ABC transporters are classified into seven families (ABC-A to -G), and among them, CFTR, a member of the ABC-C subfamily (ABC-C7), is the only ABC protein known to function as an ion channel rather than as an active transporter. CFTR's domain organization largely corresponds to that of other ABC exporters, such as Sav1866 (14), MsbA (15), and TM(287/288) (16). Two transmembrane domains (TMD1 and TMD2), each comprising six transmembrane helices, form the pore for chloride ions and are connected by cytosolic loops (CL) and extracellular loops. Two highly conserved cytosolic nucleotide binding domains (NBD1 and NBD2) can form a “head-to-tail” dimer, forming two composite binding sites for ATP at their interface. For CFTR, as for a number of other eukaryotic ABC transporters (asymmetric ABC transporters, *e.g.* all other ABC-C members, TAP1/TAP2), these ATP sites are asymmetric (17); one site is formed by consensus residues (consensus binding site or site 2), with measurable ATP hydrolysis, and the other site (degenerate binding site or site 1) contains amino acid substitutions on conserved motifs and is catalytically inactive, albeit binding ATP very tightly (18, 19). A unique CFTR domain, the so-called regulatory domain (R-domain), connects NBD1 to TMD2 and must be phosphorylated (mainly by PKA) to allow CFTR channel activity (20).

In CFTR wild-type, gating has been shown to be tightly coupled with ATPase cycles exploiting NBD dimerization (21, 22). Although the NBD dimerization for the majority of ABC transporters (exporters) is thought to induce the formation of a TMD cavity that opens toward the extracellular side for release of the transported compound (allocrite) (14), in the case of CFTR it is associated with channel opening; thus the TMDs adopt a conformation that allows selective anion flow (21, 23). It is generally accepted that phosphorylated, wild-type CFTR operates mainly through an irreversible nonequilibrium gating mechanism. Within this framework, the most frequent transitions that occur during gating involve several closed channel states and two distinct open states (21–23). In the resting state (C) the channel is closed, and ATP is bound only at site 1. After ATP binding at site 2, NBD dimerization induces opening of the channel (state O_1_). The transition from O_1_ to a second open state (O_2_) corresponds to the hydrolysis of ATP at site 2, followed by NBD dissociation and channel closure (transition O_2_ to C). The latter two transitions (hydrolysis and channel closing) constitute the hydrolytic closure pathway. In wild type, the rate of the “backward” transition O_1_ to C (also described as nonhydrolytic closure) is negligible compared with the rate of hydrolytic closure, due to the stability of the ATP-bound pre-hydrolytic state O_1_ (22, 24, 25).

For an understanding of the mechanism of CFTR, structural information on the different states and on the transitions involved in gating is crucial. A number of crystal structures are available for the isolated NBD1 and NBD2 (26–31). Given the lack of structural data on the full-length CFTR, homology modeling has been used to provide insight into the molecular details of the channel and to provide new hypotheses to be tested with experiments (32–41). Some of these homology modeling studies have been performed in combination with molecular dynamics simulations to further explore conformational states that could better represent an open channel (32, 35, 37, 38, 41) or to explore transitions from closed to open states (32). In most cases, molecular dynamics simulations showed considerable changes in the CFTR-modeled structure in a relatively short time scale (a few tens of nanoseconds). Results of molecular dynamics simulations applied on homology models should be carefully interpreted, as structural changes in a given model could derive from the instability of the model itself, especially if, as occurs for CFTR TMDs, sequence identity between modeled protein and template is low.

Homology models of putative closed state conformations of CFTR have been built using the structures of mouse P-glycoprotein (P-gp) (32) or *Vibrio cholerae* MsbA (34). The P-gp structure (Protein Data Bank code 3G5U, resolution 3.8 Å) shows a large inward-facing opening of the transmembrane cavity, and no contacts are detected at the NBD level (42). This structure was recently modified as the original database entry revealed registry shifts in several transmembrane helices (43). Thus, homology models of CFTR derived from the original structure might have incorporated the same errors. In the *V. cholerae* MsbA structure (Protein Data Bank code 3B5X), the NBDs are not engaged in a tight dimer but form contacts between residues of specific conserved motifs located at the NBD interface (15). The MsbA structure is a low resolution structure (5.5 Å) for which only the α-carbon atoms were resolved. Open state channels of CFTR have so far been modeled using the structure of Sav1866 (14) as a template. However, tight interactions on the intracellular side of the TMDs that are present in this structure might be incompatible with CFTR's channel function, blocking anion access to the cytoplasm (38, 41). The limitations associated with these templates highlight the need of new models for the closed and open states of CFTR.

Here, we applied molecular modeling techniques to build new CFTR models, and we analyzed them in the context of the CFTR gating mechanism without applying unrestrained molecular dynamics simulations. We used structural templates that, to the best of our knowledge, have not been employed before for CFTR. TM(287–288), like CFTR, is an asymmetric ABC transporter, with a degenerate and a consensus ATP-binding site. Its crystal structure (resolution = 2.9 Å) features an ATP analog bound at the degenerate site (16). ABC-B10 is a homodimeric ABC transporter crystallized in different nucleotide-bound states (resolution ranges from 2.85 to 3.30 Å) (44), with different interactions at the NBD interface compared with TM(287–288). We also built a new CFTR model using the structure of McjD (resolution = 2.7 Å), a homodimeric ABC transporter crystallized with the TMDs forming an occluded cavity that could accommodate its substrate, MccJ25, a 21-amino acid-long lasso peptide (45). Finally, we used the structure of Sav1866 as a template for an outward-facing conformation of the channel, for comparison with previously published models. Little is known about the physiological substrates of these ABC transporters, with the exception of McjD. The natural substrates of TM(287–288), ABC-B10, and Sav1866 are not known, but they all can transport a variety of molecules of different sizes and appear to have different binding sites for different molecules (16, 46–48), in common with MsbA, P-gp, and other transporters of the ABC-C subfamily (49–52).

The analysis of the transmembrane cavity of the four models suggests that only the one based on the TM(287–288) structure corresponds to a fully closed state channel. Unlike previously published closed state conformations, this CFTR model shows contacts between the two NBDs at the degenerate site, in agreement with experimental data suggesting movement (53, 54) but little separation during gating (55, 56). In addition, our analysis suggests that the McjD-based CFTR conformation could represent an open channel state better than Sav1866-based models, with openings on both sides of the transmembrane cavity. Finally, the CFTR models based on TM(287–288) and on McjD provide a possible structural underpinning for the key role of Phe-337 in determining permeation characteristics.

## Materials and Methods

### 

#### 

##### Sequence Alignments

The sequence alignments used as input to build the CFTR models were generated in sequential steps. First, we aligned the sequences of the four templates (TM(287–288), ABC-B10, McjD, and Sav1866) via a structural alignment performed with Expresso (57). Second, multiple sequence alignments for the TMDs and the NBDs were generated with MAFFT (58) using the sequences of human CFTR, TM(287–288), ABC-B10, McjD, Sav1866, *Escherichia coli* and *V. cholerae* MsbA as input sequences. For these alignments, we allowed a maximum number of 200 homologs to be retrieved by a BLAST search.

##### Model Building

Four homology models of CFTR were built with Modeler 9v12 (59), using a slow refinement protocol and 20 cycles of simulated annealing. For each CFTR conformation, 20 models were generated, and the best model was chosen based on the Discrete Optimized Protein Energy (DOPE) value and inspection of residues lining the pore and involvement in experimentally demonstrated interactions. Residues 647–845 (R-region) and residues 404–435 were not modeled because no structural templates are available for these regions of the channel.

##### CFTR Homology Models Based on Sav1866 and McjD

Following previous studies that used the Sav1866 structure to model the open state channel, during model building, distance restraints were imposed to allow experimentally proven electrostatic interactions between (i) Arg-352 and Asp-993 (60) and (ii) Arg-347 and Asp-924 (61). Additional distance restraints were also imposed to facilitate electrostatic interactions between residues (Arg-134–Glu-1104, Asp-873–Arg-933, and Arg-1102–Asp-1154) suggested to interact, based on the work of Dalton *et al.* (35). Secondary structure restraints were used to preserve the helical structure of the following segments: 81–106; 115–135; 195–216; 223–241; 301–327; 335–351; and 912–925. The same restraints were applied to build the model with McjD as a template.

##### CFTR Models Based on TM(287–288), ABC-B10

For these models, helical restraints were applied for the following segments: 81–106; 198–214; 223–231; 312–323; 347–355; and 920–930.

The NBDs of each model were derived from the homology modeling protocol, and they were not replaced with CFTR structures of isolated NBD1 monomers or homodimers because of clashes and nonoptimal side chain orientations of the residues interacting with the intracellular loops of the TMDs. The nucleotides present in the templates were taken into account during model building.

##### Model Insertion in a 1-Palmitoyl-2-oleoyl-sn-glycero-3-phosphocholine Lipid Bilayer

Each CFTR homology model was embedded in a 1-palmitoyl-2-oleoyl-*sn*-glycero-3-phosphocholine lipid bilayer following the protocol described in Corradi *et al.* (62). The simulations were carried out using GROMACS 4.5.5 (63, 64) and with the ff54a7 version of the GROMOS 96 force field (65). Each system was energy-minimized using a steepest descent algorithm, the maximum force value for convergence set to 100 kJ mol^−1^ nm^−1^. A 10-ns-long equilibration was performed with position restraints on all the heavy atoms of the protein, followed by a 15-ns-long equilibration with position restraints on the protein backbone atoms. The Protein Data Bank files of the four homology models as obtained at the end of the equilibration steps are provided in the supplemental material.

## Results and Discussion

### 

#### 

##### Sequence Alignment

Sequence alignments for homology modeling were generated in multiple steps. An initial alignment was first created only for the sequences of the templates, using Expresso (57). The sequence of human CFTR was then aligned to those of the templates using MAFFT (58), including a maximum number of 200 homologs, as described under “Materials and Methods.” The alignments for TMDs and NBDs were generated separately. For the TMDs alignment, we observed that the sequence corresponding to transmembrane helix 1 (TM1) and TM2 of McjD was not aligned to the corresponding sequences of the other three templates as in the Expresso alignment. Thus, we modified the TMD-MAFFT alignment in such a way that McjD TM1 and TM2 were aligned according to the results of the Expresso alignment. Alternative alignments are also possible, resulting in a higher degree of uncertainty with respect to TM1 and/or TM2 residue positions in the model. The alignments used are provided in the supplemental material as separate html files (TMDs.html, NBDs.html, and Templates.html), generated using the program MView (66). In these display files, only the sequences of human CFTR, human ABC-B10, Sav1866, TM(287–288), McjD, *E. coli,* and *V. cholera* MsbA are shown. Identical residues are the same color. The percentage of sequence identity with CFTR-TMD1 or CFTR-NBD1 is also indicated for each sequence.

##### Overview of the Structural Organization of CFTR Domains

**FIGURE 1.**
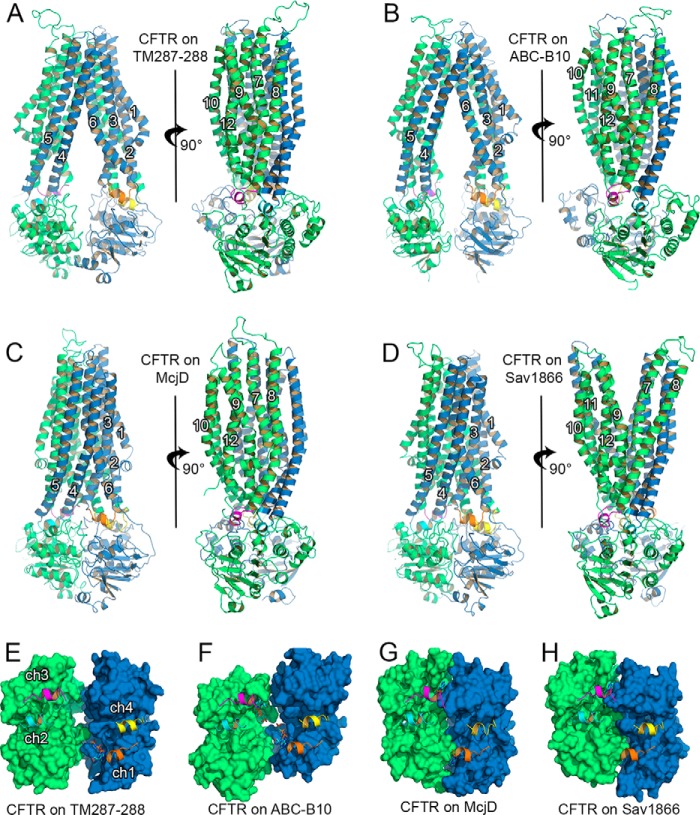
**Structural organization of the CFTR domains.** *Cartoon* representation of the homology models built using the structure of TM(287–288) (*A*), ABC-B10 (*B*), McjD (*C*), and Sav1866 (*D*). TMD1 (transmembrane helices 1–6) and NBD1 are shown in *blue*, and TMD2 (transmembrane helices 7–12) and NBD2 are shown in *green*. The TM helix number is shown in *white*. Coupling helices 1–4 are shown in *orange, cyan, magenta,* and *yellow* cartoons, respectively. *E–H,* top view of the NBDs of the model based on TM(287–288) (*E*); ABC-B10 (*F*); McjD (*G*); and Sav1866 (*H*). NBD1 is shown as *blue surface* and NBD2 as *green surface*. Coupling helices color-coded as in *A–D*. The nucleotides present in the structures used as templates are shown as *sticks*. In this orientation, the lower composite site is the degenerate site and the upper one is the consensus site.

The four homology models of CFTR presented in this paper are summarized in Fig. 1, *A–H*, and Table 1.

**TABLE 1:** **r.m.s.d. values for the four CFTR models** For TMDs, the superimposition of the models resulted in values ranging from 2.3 to 6.7 Å, when calculated on the α-carbon atoms of the TMDs and excluding the loops connecting the helical regions. The lowest r.m.s.d. values were derived from the superimposition of the two inward-facing closed state models (based on ABC-B10 and TM(287–288)) and from the superimposition of the model based on McjD with the model based on Sav1866. For NBDs, the superimposition of the models resulted in r.m.s.d. values ranging from ∼1 to 8 Å, with the lowest value corresponding to the superimposition of the models based on Sav1866 and McjD. The r.m.s.d. value for both TMDs and NBDs was calculated based on α-carbon atoms only.

	CFTR model	r.m.s.d.			
		TM(287–288)	ABC-B10	McjD	Sav1866
		Å			
TMDs	TM (287–288)				
	ABC-B10	2.6			
	McjD	6.2	6.4		
	Sav1866	6.5	6.7	2.3	
NBDs	TM (287–288)				
	ABC-B10	5.4			
	McjD	4.2	7.7		
	Sav1866	4.4	8	1.1	

As in other ABC transporters, on the cytosolic side of the TMDs, the long intracellular loops make contact with the NBDs via small helices (namely coupling helices) that sit on grooves on the NBD surface. The homologous coupling helices (Fig. 1) between TM2 and TM3 (ch1, *orange*) and between TM8 and TM9 (ch3, *magenta*) interact with the NBDs linked to the same TMD, whereas ch2 (between TM4 and TM5, *cyan*) and ch4 (between TM10 and TM11, *yellow*) sit on clefts located on the NBD linked to the opposite TMD (referred to as “domain swapping”) (14), thus allowing the TMDs to follow the association or dissociation motions of the NBDs. The models based on TM(287–288) and ABC-B10 are characterized by a similar degree of opening between the corresponding coupling helices, with the model based on TM(287–288) showing a slightly larger separation (Table 2). The models based on Sav1866 and on McjD have NBDs engaged to form a very similar tight dimer; thus, the corresponding coupling helices show the same spatial arrangement. In all the models, the distances between ch1 and ch4 and between ch2 and ch3 remain the same (Table 2).

**TABLE 2:** **Distance between coupling helices** Distances were calculated between the center of mass (defined using α-carbon atoms only) of the coupling helices. The *italic type* indicates those coupling helices whose distance remains unchanged across the models.

CFTR model	Distance between coupling helices					
	1–2	1–3	1–4	2–3	2–4	3–4
	Å					
TM(287–288)	37	37	*13*	*13*	41	37
ABC-B10	31	30	*13*	*12*	36	32
McjD	24	26	*12*	*12*	28	26
Sav1866	24	26	*13*	*12*	27	25

**FIGURE 2.**
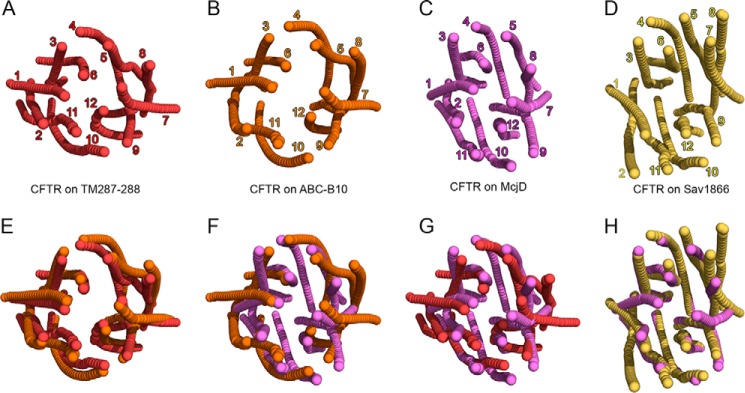
**Transmembrane helix axes.** *A–D,* view from the extracellular side of the helix axes of the CFTR model based on TM(287–288), *red* (*A*); ABC-B10, *orange* (*B*); McjD, *violet* (*C*); and Sav1866, *yellow* (*D*). *E–H,* superimposition of the transmembrane helix axes of different CFTR models color-coded as in *A–D*.

The transmembrane region of the helices creates transmembrane cavities of different volume among the models. Fig. 2 shows the axes of the TMD helices, including their extension on the cytosolic and extracellular side, calculated using the program HAXIS (67). Both the TM(287–288)- and the ABC-B10-based models are inward-facing, with the cavity wide open toward the intracellular side. However, the cavity upper side is narrower in the TM(287–288)-based model due to the different bending and kink of some of the transmembrane helices. The kink of both TM5 and TM11 is clearly different in the two models (Fig. 2, *A versus B*), although TM4 and TM10 appear more curved, and the extracellular ends of TM6 and TM12 are more distant in the ABC-B10-based model, thus creating a widening in the upper side of the cavity (Fig. 2*E*). The models based on Sav1866 and McjD, although characterized by a very similar structural organization on the intracellular side, display significant differences in the TMDs (Fig. 2*H*). In the model based on McjD (Fig. 2*C*), the extracellular ends of the helices resemble those of the inward-facing models, whereas in the Sav1866-based one (Fig. 2*D*), TM1–2 and TM7–8 are completely separated from other helices of the same TMD.

The TM(287–288)- and ABC-B10-based models, although showing a similar degree of opening between the coupling helices, have distinct arrangements of their NBDs, as demonstrated by the difference in r.m.s.d. values obtained from superposition with tight NBD dimer structures (Table 1). The NBDs in the TM(287–288)-based model are partially open, with contacts mainly detectable at the degenerate site (Fig. 1*E*, *lower site*). In the ABC-B10 based model, the two NBDs appear translated in opposite directions, such that the grooves for the binding of the domain-swapped coupling helices are in line with each other (Fig. 1*F*), although in the other three models they lie on parallel lines. The tight NBD dimer of the McjD-based and the Sav1866-based models are almost equivalent, with an r.m.s.d. value of ∼1 Å (Table 1 and Fig. 1, *G* and *H*).

Substituted cysteine accessibility mutagenesis studies aiming at identifying residues contributing to the CFTR pore have been performed on several transmembrane helices. Although the results on individual residues might differ, all studies agree on a pore architecture consisting of the following three regions: an outer vestibule, a narrow region, and a wider inner vestibule (37, 68–74). We will first focus on the narrowest region whose structure has crucial consequences on the permeation of water and ions through the four models proposed (74–78).

##### Narrow Region and Inner Vestibule

**FIGURE 3.**
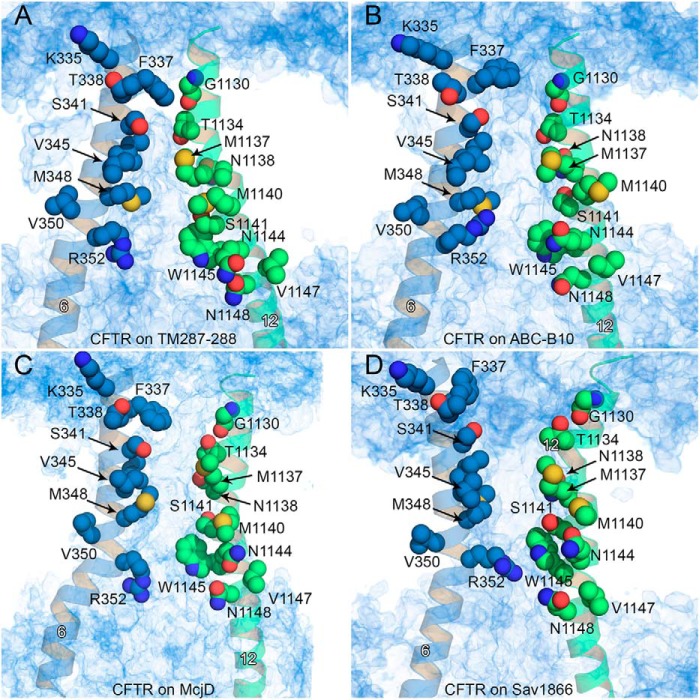
**Water density within the pore.** *A–D,* number density of water molecules is shown as a *blue* volume map for the model based on TM(287–288) (*A*), ABC-B10 (*B*), McjD (*C*), and Sav1866 (*D*). TM6 and TM12 are shown as *blue* and *green cartoons*, respectively, and selected residues along these helices are shown as *spheres* for reference. The helix number is shown in *white*.

**FIGURE 4.**
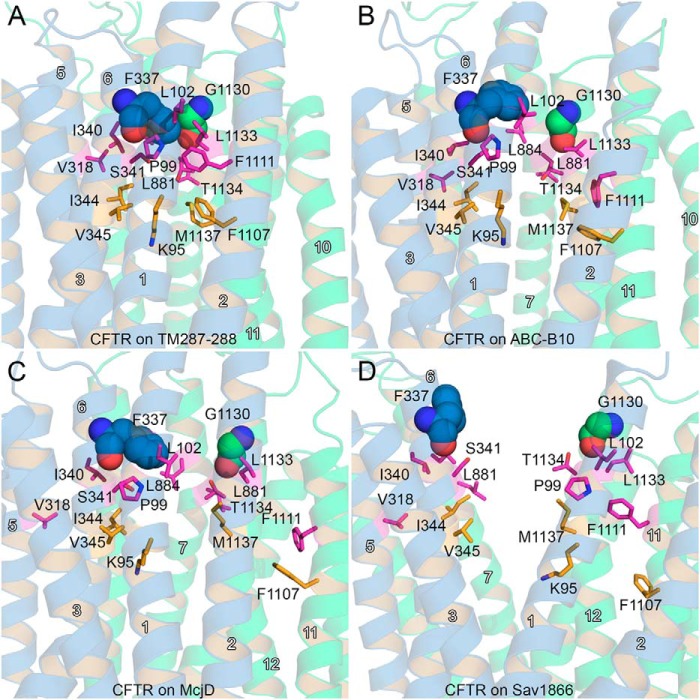
**Narrow region of the pore around Phe-337.** *A–D,* structures of the CFTR model based on TM(287–288) (*A*), ABC-B10 (*B*), McjD (*C*), and Sav1866 (*D*) obtained at the end of the equilibration steps are shown as *blue* (TMD1) and *green* (TMD2) cartoons. Residues forming a first constriction from the intracellular side in the TM(287–288)-based CFTR model (*A*) are shown as *orange sticks*. Hydrophobic residues surrounding Phe-337 are shown as *magenta sticks*. This constriction is not present in the ABC-B10 model (*B*), but in the McjD-based model (*C*), it allows water molecules to flow through. The helix number is shown in *white*.

**FIGURE 5.**
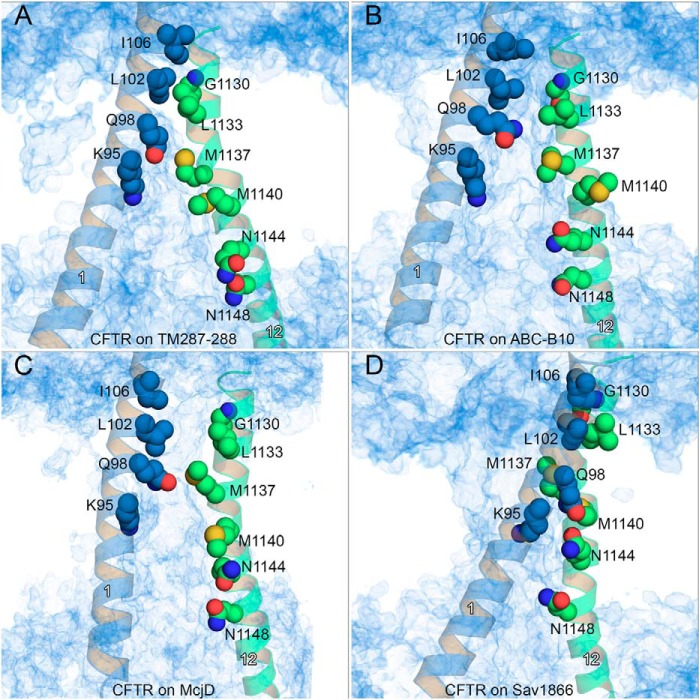
**TM1 and TM12 residues in the narrow region of the pore.** *A–D,* water density is shown as a *blue* volume map for the model based on TM(287–288) (*A*), ABC-B10 (*B*), McjD (*C*), and Sav1866 (*D*), as shown in Fig. 3. TM1 and TM12 are as *blue* and *green cartoons*, respectively, using the same orientation as in Fig. 3. Selected residues along these helices are shown as *spheres* for reference. The helix number is shown in *white*.

**FIGURE 6.**
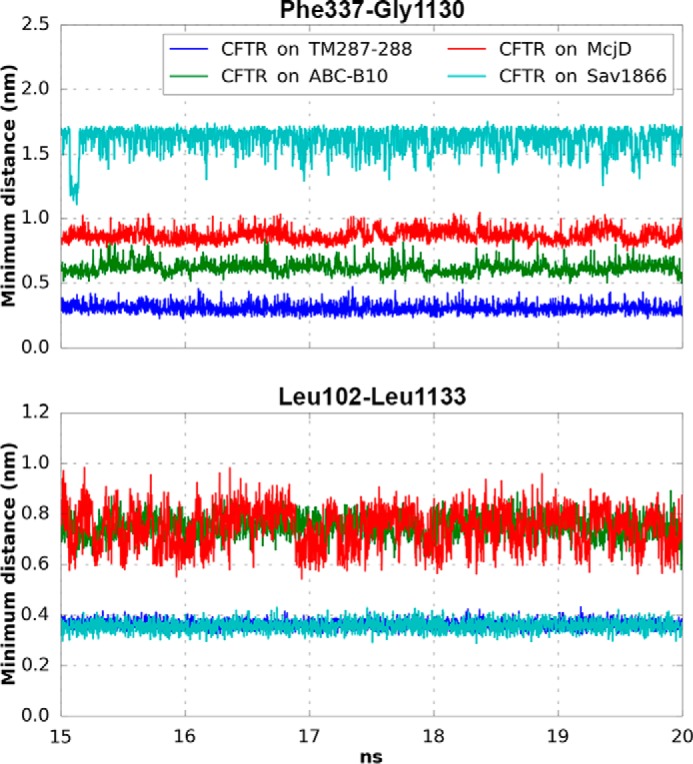
**Minimum distance between Phe-337 and Gly-1330 and between Leu-102 and Leu-1133.** The minimum distance between Phe-337 and Gly-1130 (*upper panel*) and between Leu-102 and Leu-1133 (*lower panel*) was calculated for each model during the last 5 ns of the equilibration performed with no position restraints on the protein side chains.

Residues of TM6 have been shown to be critical for both anion permeation and channel gating (72), with residues Phe-337–Ser-341 forming the narrow portion of the pore (68, 72, 79–81), and a number of TM6 residues up to Gln-353 lining the permeation pathway further toward the cytosol (68, 72). In addition, I336K and T338I are known among CFTR disease-causing mutations (82, 83). In the four homology models presented in this work, the extracellular ends of TM6, as well as those of TM1, TM11, and TM12 (68–74), are critical in determining the degree of opening of the channel, thus affecting the permeation of water molecules deeper into the pore. For three of our models (based on TM(287–288), ABC-B10, and McjD), the narrow region of the pore corresponds to residues Phe-337, Thr-338, and Ser-341 of TM6, which are aligned to residues Gly-1130–Ile-1133 of TM12, and Leu-102–Ile-106 of TM1 in agreement with experimental studies (69, 74). In this context, we analyzed how the position of Phe-337 (TM6 (79, 80)) in the individual models altered the dynamics of water molecules in the channel. Possible permeation pathways were described based on water density calculations performed for the last 5 ns of the equilibration steps, and the degree of opening of the channel was estimated based on distances observed in simulations at the most restricted regions in these four CFTR models (Figs. 3–6).

In the TM(287–288)-based model, Phe-337 is located in the narrowest region of the pore, and it operates as a gate to block the flow of water molecules from the extracellular side to the intracellular side, and vice versa (Fig. 3*A*). Immediately below, a cluster of mainly hydrophobic residues such as Pro-99 (TM1), Leu-102 (TM1), Ile-340 (TM6), Ser-341 (TM6), Leu-881 (TM7), Phe-1111 (TM11), Leu-1133 (TM12), and Thr-1134 (TM12) hampers, possibly by forming a “mainly hydrophobic gate” (see below and Ref. 84), the upward movement of the solvent molecules (Fig. 4*A*). In addition, their bulk limits the flexibility of Phe-337, contributing to closure of the gate. Further on the cytosolic side, a narrow internal vestibule is formed by the side chains of Lys-95 (TM1), Ile-344 (TM6), Val-345 (TM6), Phe-1107 (TM11), and Met-1137 (TM12).

In the ABC-B10-based model, Phe-337 is still located in a narrow region of the pore. However, because of the different curvature of the TM helices and to the openings on the extracellular side, Phe-337 does not form a gate, as streams of water molecules moving from the extracellular side to the intracellular one, and vice versa, can be detected (Fig. 3*B*). Because of the different curvature of the transmembrane helices, the cluster of hydrophobic residues located on the intracellular side of Phe-337 does not form a constriction as in the TM(287–288)-based model (Fig. 4*B*).

In the CFTR model based on McjD, Phe-337 is again positioned in the constricted part of the pore (Figs. 3*C* and 4*C*). The cavity widens on the intracellular side after the clusters of mainly hydrophobic residues mentioned above. The equilibration performed with no restraints on the side chains delineated a channel with asymmetric shape, which again allowed water molecule flow in both directions. Again, Phe-337 is the residue that mainly controls the permeation through the narrow region of the pore (Fig. 3*C*). Thus in the ABC-B10- and McjD-based models, Phe-337 adopts a conformation in which it appears to behave more as a filter than a gate, as described for open state channels (79, 80, 85). In the CFTR model based on Sav1866, the large separation between the two “wings” (TM1, -2, and -9–12 and TM3–8) forms a wide extracellular cavity with no narrow region near Phe-337 (Figs. 3*D* and 4*D*), with no suggestion of a filter/gate role for such a residue.

The presence of a cluster of mainly hydrophobic residues around a given residue that, like Phe-337, can act as a gate or as a filter is not unprecedented in channel proteins. For the nicotinic acetylcholine receptor, computational studies identified a central region in the pore, with a radius of ∼3 Å, where a cluster of hydrophobic residues act as a gate to block ion permeation (84). In a homologous bacterial pentameric ligand-gated ion channel, the bulky side chains of isoleucine residues form a constriction in the structure of the pore, whose level of hydration changes as a function of the conformational state (86). For CFTR, Phe-337 may play a similar role by completely occluding the cavity in the TM(287–288)-based model and by letting water (or anion) molecules flow through the channel in other conformational states. Together, Phe-337 and the cluster of hydrophobic residues near this position likely determine the lyotropic selectivity sequence by forcing permeating ions to shed their hydration shell (80, 87).

In addition to Phe-337, residues of TM1 in close proximity with residues of TM12 also contribute to control pore accessibility. In particular, it is known that the modification of a cysteine residue at the position corresponding to Leu-102 (TM1) has strong effects on permeation (74). In addition, residues of TM1 from Leu-102 to Leu-106 are described as belonging to a narrow region of the pore as well (74). In our models, the Leu-102 side chain is at the same level as Gly-1130 and Leu-1133 of TM12 (Fig. 5). To compare the degree of opening of the CFTR models near Phe-337, we measured the minimum distance between Phe-337 and Gly-1130 and between Leu-102 and Leu-1133 during the last 5 ns of the equilibration performed with no restraints on the residues' side chains (Fig. 6). Interestingly, the side chains of Leu-102 and Leu-106 are in close contact with residues of TM12 (Gly-1130 and Leu-1133) in the closed CFTR conformation based on TM(287–288). The separation increases for the model based on ABC-B10 and on McjD, respectively. For the outward-facing model of CFTR based on Sav1866, the average minimum distance between these two residues corresponds to that of the closed-state model.

It has been suggested that to account for permeation characteristics of anion flow through the CFTR pore, an opening of at least 5.3 Å should be present (88, 89). Based on this criterion, only the model based on TM(287–288) could be described as a fully closed state channel (Fig. 6). The model based on McjD, with an average minimum distance of 8.5 and 7 Å for Phe-337–Gly-1130 and Leu-102–Leu-1133, respectively, corresponds to a hydrated state and could potentially represent an open state channel conformation. An open state assignment for the McjD-based model is also consistent with the tightly dimerized NBDs (21). Both the water distribution analyses (see above, Figs. 3*B* and 5*B*) and minimum Phe-337–Gly-1130 and Leu-102–Leu-1133 distances (average 6.2 and 7.5 Å, respectively, Fig. 6) suggest that the CFTR conformation corresponding to the model based on ABC-B10 is also a hydrated state, due to the larger openings created by the transmembrane helices on the extracellular side of the membrane. However, because in such a model the NBDs are not forming a tight dimer, it likely does not reflect a conformation that wild-type CFTR can frequently adopt. Further studies are required to determine whether a similar conformation is visited, in particular by mutants that are known to form open channels despite drastic alterations of the NBD dimer interface that would make tight dimer formation impossible (*e.g.* G551D (90) or constructs lacking NBD2 (91)).

Figs. 3 and 5 also highlight additional residues of TM1, TM6, and TM12 that are exposed in the channel cytosolic vestibule. In agreement with experimental data (68–70), the pore is lined by Ile-344, Val-345, Met-348, Ala-349, Arg-352, and Gln-353 of TM6 and Asn-1138, Met-1140, Ser-1141, Gln-1144, Trp-1145, Asn-1148, and Ser-1149 of TM12. Cysteine residues replacing Phe-337, Thr-338, Ser-341, Arg-347, or Arg-352 reduce single channel current (72). In the McjD-based model, Phe-337, Thr-338, Ser-341, Arg-347, and Arg-352 directly line the pore, with Phe-337, Thr-338, and Ser-341 located in the narrowest region (Fig. 3). Cysteine replacement side chains at these positions are likely to disrupt optimal permeation conditions by disrupting crucial sites of interaction between permeating anions and the pore walls and/or by removing positive charges involved in structurally important salt bridges (60, 61).

##### Outer Vestibule

Experimental studies proposed that charged residues such as Arg-334, Lys-335 (TM6), Arg-104 (TM1), and Arg-117 (TM2) might help concentrate extracellular Cl^−^ anions in proximity of the pore (75, 76, 78). Among them, Arg-334 and Lys-335 play an important role in determining permeation properties of pore, with Arg-334 to Trp being a mutation causing mild forms of cystic fibrosis (76). In addition to charged residues, uncharged amino acids have been proposed to contribute to the architecture of the outer vestibule, in particular Ile-106, Ala-107, and Tyr-109 (74).

**FIGURE 7.**
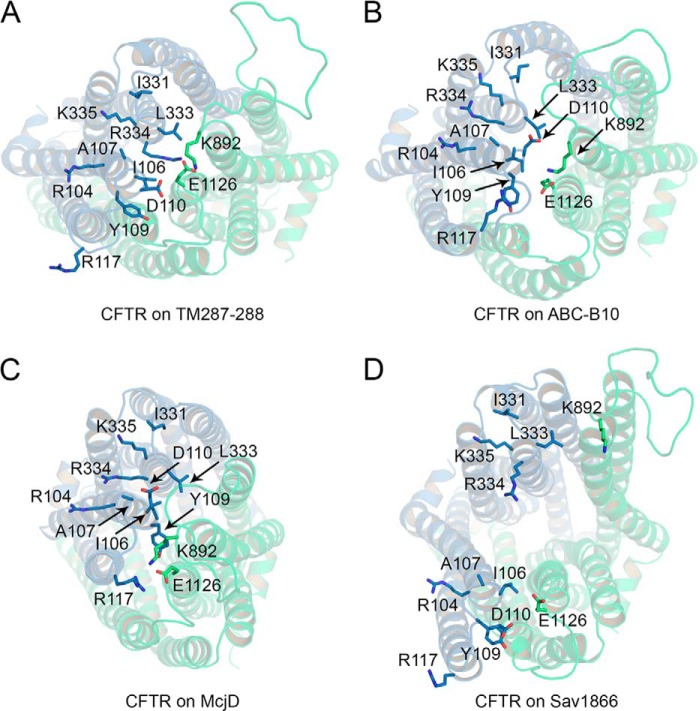
**Outer mouth of the pore.** *A–D,* top view of the CFTR models based on TM(287–288) (*A*), ABC-B10 (*B*), McjD (*C*), and Sav1866 (*D*). Residues contributing to closing the cavity on the extracellular side of the TM(287–288)-based model and additional residues contributing to the outer vestibule are shown as *sticks*.

In broad agreement with this experimental evidence, in the TM(287–288)-based model the outer side of the pore is lined by residues of TM1, TM6, TM7, and TM12 (Fig. 7*A*). At the extracellular end of these transmembrane helices, the side chains of Ile-106, Tyr-109, Asp-110 (TM1), Leu-333 (TM6), Lys-892 (TM8), and Glu-1126 (TM12) contribute to reducing the exposure to the extracellular environment of the residues below. These same residues contribute to the narrowing of the cavity toward the extracellular side in the ABC-B10- and in the McjD-based models (Fig. 7, *B* and *C*). The outward-facing conformation of the CFTR model based on Sav1866, however, shows a large opening on the extracellular side (Fig. 7*D*), with no obvious separation between “outer mouth” and pore.

**FIGURE 8.**
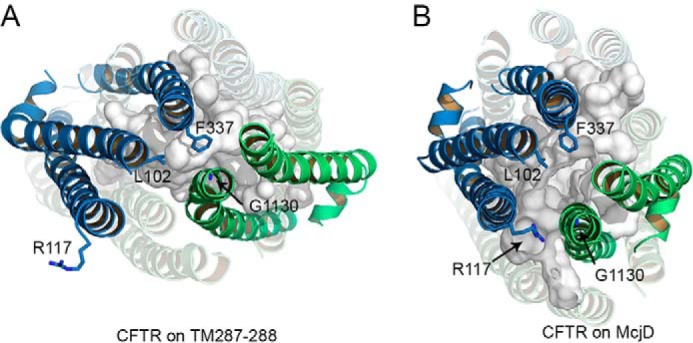
**Outer-mouth of the pore and cavity shape.** *A* and *B,* top view of the main transmembrane cavity (*white transparent* surface) of the CFTR models based on TM(287–288) (*A*), and McjD (*B*). The extracellular end of the transmembrane helices completely closes the cavity in the closed state model (*A*), although an opening is detected in the open state model (*B*). TM1, -2, and -6 of TMD1 and TM7 and -12 of TMD2 are shown as *blue* and *green cartoons*, respectively. The remaining transmembrane helices are shown as *transparent cartoons*. Leu-102, Phe-337, and Arg-117 of TMD1 are shown as *blue sticks*, and Gly-1130 of TM12 is shown as *green sticks*.

Fig. 8 highlights the main internal cavity shown as a *white transparent* surface. In the TM(287–288)-based model, the cavity is clearly closed by the constriction located near Phe-337, but in the McjD-based model, the cavity opens up toward the extracellular space. However, extracellular to the narrowest region of the pore (Phe-337), the cavity opens more toward TM2, TM11, and TM12. This observation might explain why mutations at Arg-117 in TM2 caused inward rectification and a decrease in single channel conductance, consistent with the R117H mutation causing mild forms of cystic fibrosis (76, 78).

##### Cytosolic Intracellular Loops and Lateral Opening

In ABC transporters, the region at the interface between TMDs and NBDs, *i.e.* the transmission interface, includes part of the CLs, characterized by the presence of the coupling helices. These helices provide a link relaying conformational changes from the NBDs to the TMDs (14, 15). Mutations affecting this interface (including the commonest CF mutation, deletion of Phe-508) alter folding, stability, and thus function of the transporters (92–95). In MRP1, also a member of the ABC-C subfamily (ABC-C1), residues of the CL between TM10 and TM11 in TMD2 (the CL comprising ch4 in CFTR) are important for the folding and the transport mechanism (93). This region is highly conserved among ABC-C transporters, thus suggesting similar roles for the corresponding residues in CFTR and other members of this subfamily. Furthermore, studies performed on ABC-C6 showed that some mutations causing pseudoxanthoma elasticum, a genetic disorder that affects elastic tissues, also map to the transmission interface (94).

Several loops of the NBDs interact with the CLs. Among them, the X-loop, seven amino acids preceding the signature motif, is thought to sense changes in the NBDs and thus is likely an important functional part of the transmission interface. In TAP, for example, an asymmetric and heterodimeric ABC-B transporter, the TAP2 X-loop is involved in the cross-talk between domains (95). Computational studies on ABC transporters also highlighted the nucleotide-dependent interactions of the X-loop glutamate with the CLs (96–99).

**FIGURE 9.**
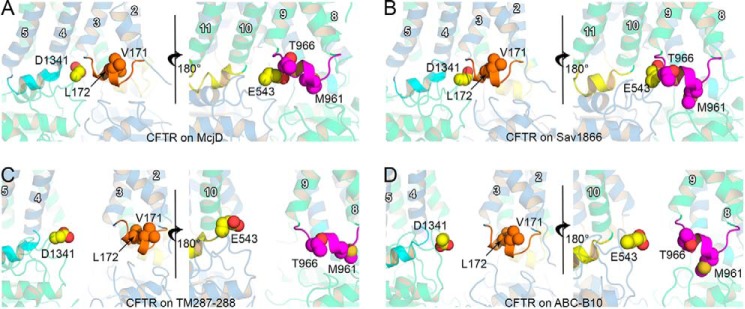
**Transmission interface.** In the CFTR model based on McjD (*A*) and on Sav1866 (*B*), the relative orientation of the intracellular loops bearing the coupling helices and the NBDs with the X-loop motifs is very similar. Because of the dimerized NBDs, the conserved X-loop residues (NBD2-Asp-1341 and NBD1-Glu543, *yellow spheres*) are within cross-linking distance with residues of ch1 (*orange*) and ch3 (*magenta*). In the CFTR model based on TM(287–288) (*C*) and on ABC-B10 (*D*), the X-loop residues are not interacting with the coupling helices of the opposite TMD.

Cross-linking experiments confirmed for CFTR the “domain swap” pattern of interactions between the NBDs and the CLs revealed by the Sav1866 crystal structure (100). The models based on McjD and Sav1866 share the same structural arrangement of the CLs, with the conserved negatively charged residues of the X-loop within cross-linking distance of residues of the coupling helix of the opposite half (Fig. 9, *A* and *B*). In contrast, in both CFTR inward-facing models proposed in this work, no contacts are detected between ch3 and NBD1-Xloop or ch1 and NBD2-Xloop (Fig. 9, *C* and *D*).

So far, one of the main problems of open channel CFTR models based on Sav1866 has been the lack of an opening on the intracellular side, which would allow Cl^−^ ion movement between the pore-like transmembrane cavity and the cytosol (34, 38, 41, 101). The same problem could affect the CFTR model based on McjD. It should be noted that the domain-swapped interaction between the X-loops in the NBDs and the coupling helices does occur during the gating cycle of CFTR (36, 100). Because channel opening corresponds to formation of a tight dimer interface (21), it is likely that the tightly packed NBD/TMD interface seen in the Sav1866 crystal structure is present in open channel conformations and therefore the permeation of Cl^−^ ions to and from the cytosol might occur through different regions of the protein.

**FIGURE 10.**
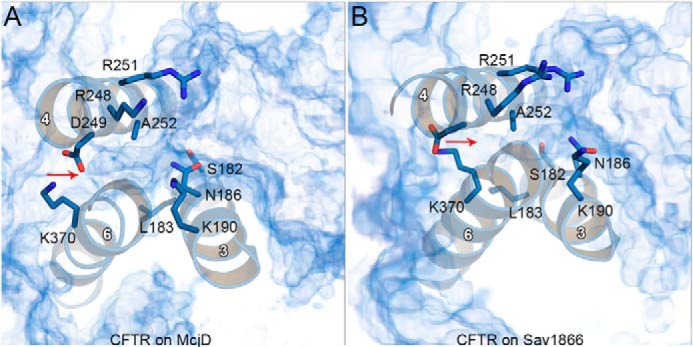
**Lateral openings in the intracellular side of the channel.** Top view of the larger opening identified on the CFTR model based on McjD (*A*). The opening is highlighted by a *red arrow*, and surrounding residues of TM3 (Ser-182, Leu-183, Asn-186, and Lys-190), TM6 (Lys-370), and TM4 (Arg-248, Asp-249, and Arg-251 oriented toward TM5, not shown, Ala-252) are shown as *sticks*. The water density is shown as *blue* volume map. The corresponding region in the CFTR model based on Sav1866 is shown in *B*.

In the CFTR model based on McjD, the water density analysis also revealed a lateral opening located between the cytosolic ends of TM6 and TM4 (Fig. 10*A*), which is not present in the Sav1866-based model (Fig. 10*B*). On the cytosolic side, this portal is lined by Lys-370, Asp-249, Ala-252, Gly-253, and Ser-256. Inside, Lys-190, Arg-248, Asn-186, Ser-182, Asn-189, and Ile-255 delineate the tunnel. A number of positively charged residues line the inside of the cavity, among them Arg-248, Arg-303, and Arg-352, and could potentially funnel Cl^−^ ions in and out of the lateral opening (Fig. 10). It is interesting to note that both Arg-352 and Arg-303, and, very recently, also Lys-190, Arg-248, and Lys-370 have been shown to modulate conduction through CFTR, by concentrating permeating anions in the intracellular mouth of the pore (102, 103). Consistent with these experimental data, our McjD-based model shows that the tight interaction between CLs and NBDs does not necessarily create a sealed cavity for water and small ions and that different bending and curvature of the helices in the intracellular side might be crucial to forming lateral openings. A recent modeling study, where an open state channel was built by means of molecular dynamics simulations applied to an initial Sav1866-based model, also highlighted the possibility of lateral tunnels in an open state channel model (41) (the lateral opening between TM6 and TM4 described here for the McjD-based model largely corresponds to the “the secondary lateral tunnel” described by Mornon *et al.* (41)).

Despite the overall agreement between our models and the experimental data described above (102, 103), the electrophysiological observation of voltage-dependent open channel block by several relatively bulky pore blockers (*e.g.* intracellularly applied glibenclamide and 5-nitro-2-(2-phenylpropylamino)benzoate (104, 105)) is hard to reconcile with the presence of narrow lateral tunnels in the open CFTR channel. Despite their bulk, the blockers can freely access their binding site, likely located around Lys-95, in the channel open state. One clear limitation of CFTR homology modeling so far has been the inability to accurately model the R-domain, due to the absence of homologous structures in the available crystallized proteins. It is therefore tempting to speculate that the lateral openings in CFTR are wider than those seen in our models, due to the presence of the R-domain. Intriguingly, the appearance of the R-domain in CFTR's evolutionary lineage appears to occur very close in time with the acquisition of the novel channel function (106).

**FIGURE 11.**
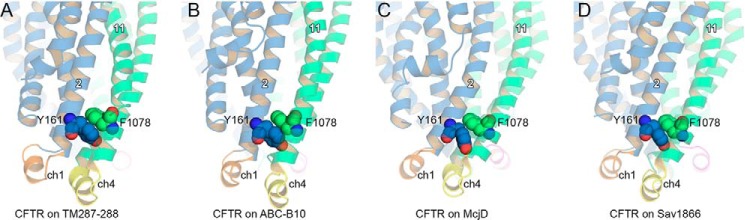
**Conserved aromatic residues in the intracellular loops.** Tyr-161 of TM2 and Phe-1078 of TM11 form a pair of aromatic residues that are highly conserved among ABC transporters. *A–D*, CFTR model based on TM(287–288) (*A*), ABC-B10 (*B*), McjD (*C*), and Sav1866 (*D*). TMD1 and TMD2 are shown as *blue* and *green cartoons*, respectively. Coupling helices 1 and 4 are shown in *orange* and *yellow*, respectively. The NBDs are not shown for clarity.

An additional feature of the CLs concerns TM2 and TM11. Our previous work on TAP identified pairs of aromatic residues in TAP1-TM2 and TAP2-TM5 and in TAP1-TM5 and TAP2-TM2 that are conserved among ABC-B members and bacterial exporters (62). Literature data available for TAP1-Phe-265 (TM2) suggested a structural role of this residue in potentially stabilizing the overall structure of the cavity, thus affecting the kinetics of transport (107). Based on the analysis of the TAP models and comparisons with homologous transporters, our modeling studies further supported the hypothesis that aromatic residues at these positions could be involved in the correct packing of the intracellular ends of the transmembrane helices. Interestingly, we observed here that ABC-C transporters also show a pair of conserved aromatic residues corresponding to those previously identified for other transporters. In the case of CFTR, the residues are Tyr-161 of TM2 and Phe-1078 of TM11 (114), and in the CFTR models proposed in this work, they are oriented as seen previously for TAP and homologous transporters (Fig. 11). Given the low sequence identity between ABC-B and ABC-C transporters, the presence of conserved aromatic residues at given locations in the cytosolic side of TM2 and TM11 (TM5 in homo- and heterodimers) strengthens the hypothesis of a possible role in stabilizing inter-helix interactions.

##### Opening Transition

**FIGURE 12.**
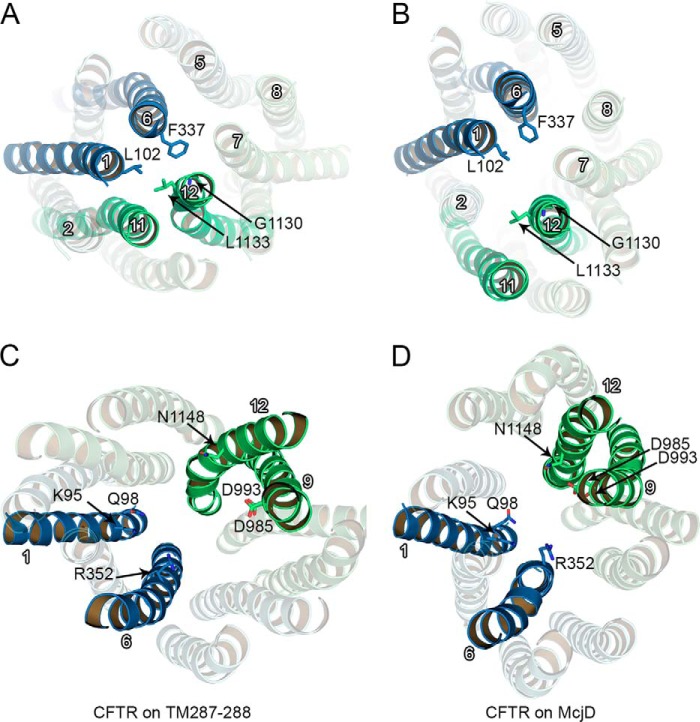
**From closed to open channel.** *A* and *B,* top view of the CFTR model based on TM(287–288) (*A*) and McjD (*B*) here described as a closed and an open state channel, respectively. In the closed state (*A*), the extracellular ends of TM1, -2, -6, and -5 (TMD1) and TM7, -8, -11, and -12 (TMD2) are distributed along two hypothetical parallel lines, forming the interface between TMD1 and TMD2. Upon channel opening (*B*), this alignment shifts, thus allowing access to the pore. *C* and *D,* view from the intracellular side of the transmembrane cavity of the closed (*C*) and open (*D*) models. TMD1 and TMD2 are shown in *blue* and *green cartoons*, respectively. *A* and *B*, Leu-102, Phe-337, Gly-1130 and Leu-1133, contributing to the narrow region of the pore, are shown as *sticks. C* and *D*, functionally important residues on the intracellular side of the cavity (Lys-95, Gln-98, Arg-352, Asp-985, Asp-993, and Gln-1148) are shown as *sticks*.

For CFTR, changes in the accessibility of specific residues during channel gating have often been interpreted in terms of the transporter-alternating access mechanism, assuming different tilting of the helices of the TMDs in open and closed states (74, 101). However, a simple transition from an inward-facing conformation to a Sav1866-like outward facing conformation is inconsistent with much of the available experimental data (108). Fig. 12 shows a comparison of the closed (TM(287–288)-based) and open (McjD-based) state models presented in this work. During channel opening, driven by NBD dimerization, the wide cytosolic side of the cavity formed by the TMDs is closed off, inducing changes in the tilting of the transmembrane helices, which on the extracellular side straighten and on the intracellular side form a tight tetrahelix bundle (96, 109). On the extracellular side, in both conformations, the ends of TM3–4 and TM9–10 are removed from the TMD1/TMD2 interface, facing the membrane lipids, although at the interface, the extracellular ends of TM2, 1, 6, and 5 (from TMD1) and TM8, 7, 12, and 11 (from TMD2) are approximately aligned on two parallel lines. Upon channel opening, the aligned helices slide along the interface, while simultaneously the extracellular ends of TMD1 and TMD2 separate slightly, opening a central passage toward the deeper transmembrane cavity (Fig. 12, *A* and *B*).

We do not observe significant rotations of individual helices around their axes (note that the orientation of selected residues such as Phe-337 in TM6 and Leu-1133 in TM12 is similar in both models, Fig. 12, *A* and *B*). However, the structural changes the models predict could alter the exposure of specific residues due to modifications of the helix/helix interfaces, in a way consistent with many of the state-dependent accessibility changes experimentally observed. In particular, upon opening, TM1 has been suggested to undergo conformational changes that expose previously buried residues to the pore cavity (74, 110) and approximate it to TM6 (111). Fig. 12 shows how TM1, buried behind TM6 and TM11 in the closed state model, lines the pore cavity in the open state model, moving closer to TM6, whereas TM6 and TM12 move apart from each other (72, 74, 111). Conformational changes suggesting a helix rotation in TM6 were inferred based on changes in gating characteristics following covalent modification of, among others, a cysteine introduced at position 352. A significant increase in open probability was observed only when a smaller, positively charged reagent was used (72). To rationalize the effects of small, positively charged reagents when a cysteine replaces Arg-352 (72), we can hypothesize that the shorter adduct (-CH_2_CH_2_-NH_3_^+^) on the cysteine might restore a functionally important positive charge in a position similar to that present in the native arginine, whereas the bulkier adduct (-CH_2_CH_2_-N(CH_3_)_3_^+^) might also sterically destabilize the McjD-like open conformation, given the relative narrowing of this region of the pore.

In addition, a reduced accessibility for TM12 residues such Asn-1148 was observed upon channel opening (73, 85). In the TM(287–288)-based model, this side chain is exposed to the wide cytoplasmic cavity, whereas in the McjD-based one it is facing the pore, but deep within the intracellular vestibule, past the narrow lateral tunnels, and in close proximity of negatively charged Asp-993 and Asp-985 (Fig. 12, *C* and *D*). A transition from a TM(287–288)-like to a McjD-like conformation in CFTR would thus likely predict a decrease in reactivity of a thiolate at position 1148 both toward bulky cytosolic reagents (73) and toward smaller permeant probes (85). The need to negotiate relatively tight lateral tunnels on the cytosolic permeation pathway to gain access to sites deep within the inner vestibule might explain the “paradoxical” observation that the inner vestibule appears to become less accessible upon channel opening (73, 112).

##### Conclusions

In this work, we explored possible conformational states visited during the CFTR gating cycle by means of homology modeling techniques. We used the crystal structure of TM(287–288), which, like CFTR, is an asymmetric ABC transporter, ABC-B10, McjD, and Sav1866 as templates, and we investigated the structural properties of the transmembrane cavity. Overall, the models show a structural organization in which the outer mouth of the channel (Figs. 7 and 8), the narrow region of the pore (Figs. 3 and 5), and the intracellular loops (Figs. 9–11) can be related to experimental data. Our findings suggest that the conformation based on TM(287–288) represents a closed-state model, with a major role of Phe-337 as a gating residue in blocking solvent exchange between the extracellular and intracellular side of the channel. Consistent with this interpretation, very recent studies using a channel-permeant thiol reagent identify the narrowest region of the pore of CFTR as constituting the gate (85). The CFTR model based on McjD displayed a continuous transmembrane permeation pathway as follows: open toward the extracellular side via a narrowing controlled by Phe-337 and open toward the cytosol via lateral openings bypassing the tight TMD/NBD interface. At the present time, we cannot establish whether such a hydrated channel-like conformation corresponds to a fully conductive state or an intermediate state toward a fully open state channel. However, we observed more features in line with an open state channel for the model based on McjD than for the model based on Sav1866.

CFTR's C → O_1_ opening transition might therefore represent a transition from a TM(287–288)-like to an McjD-like conformation. In this scenario, in the inward-facing TM(287–288) conformation, CFTR would retain a functional extracellular gate (Figs. 3–5*A*), homologous to the one preventing diffusion to the extracellular space of allocrites bound to *bona fide* transporters. However, in the outward occluded conformation, the “broken” ABC exporter CFTR (113) would have diverged from its transporter relatives to allow the flow of small anions past structures corresponding to both the intracellular (Fig. 10) and extracellular (Figs. 3–5*C*) gates preventing allocrite movement. In a similar way, in CFTR conformations related to the outward-facing Sav1866 conformation, anions might bypass, via lateral openings (41), the tight TMD/NBD interface, comprising the vestigial intracellular gate (preventing passive allocrite diffusion back toward the cytosol, during ATP hydrolysis-coupled export). Further studies are needed to establish whether the McjD-like conformation described here could indeed represent CFTR's pre-hydrolytic O_1_ open state, occupied for most of the normal, hydrolytic open burst.

## Author Contributions

P. V., V. C., and D. P. T. conceived the study. V. C. carried out the homology modeling. V. C. and P. V. analyzed and interpreted the models. V. C. and P. V. wrote the paper with input from D. P. T. All authors reviewed the results and approved the final version of the manuscript.

## Supplementary Material

Supplemental Data

## Footnotes

3 The abbreviations used are:

CFTR: cystic fibrosis transmembrane conductance regulator
TMD: transmembrane domain
ABC: ATP-binding cassette
r.m.s.d.: root mean square deviation
TM: transmembrane
CL: cytosolic loop
P-gp: P-glycoprotein.
