# Supplementary material for: Cystic Fibrosis Transmembrane Conductance Regulator (CFTR): CLOSED AND OPEN STATE CHANNEL MODELS
Source: J Biol Chem. 2015 Jul 30;290(38):22891–906. doi: 10.1074/jbc.M115.665125 (PMC4645605; doi:10.1074/jbc.M115.665125)
Supplement: Supplemental Data [file supp_290_38_22891__index.html]

Cystic fibrosis transmembrane conductance regulator (CFTR): closed and open state channel models — Cystic Fibrosis Transmembrane Conductance Regulator (CFTR) — CFTR: Open and Closed State Models — Supplemental Data 

# Cystic Fibrosis Transmembrane Conductance Regulator (CFTR)

## Supplemental Data

- Sequence alignments, pdb files (.zip, 606 KB) - Sequence alignments and coordinate files for the homology models described in the paper.
