# Supplementary material for: Cystic Fibrosis Transmembrane Conductance Regulator (CFTR): CLOSED AND OPEN STATE CHANNEL MODELS
Source: J Biol Chem. 2015 Jul 30;290(38):22891–906. doi: 10.1074/jbc.M115.665125 (PMC4645605; doi:10.1074/jbc.M115.665125)
Supplement: Supplemental Data [file supp_M115.665125_jbc.M115.665125-1.zip › NBDs.html]

# 

|  |
| --- |
| ``` Reference sequence (1): ABCC7_01 Identities normalised by aligned length. Colored by: property ``` |
| ``` 1 ABCC7_01   100.0%  GFGELFEKAKQNNNNRKTSNGDDSLFFSNFSLLGTPVLKDINFKIERGQLLAVAGSTGAGKTSLLMVIMGELEPSEGKIKHSGR-------------ISFCSQFSWIMPGTIKENIIFGV-----SYDEYRYRSVIKACQLEEDISKFAEKDNIVLGEGGITLSGGQRARISLARAVYKDADLYLLDSPFGYLDVLTEKEIFESCVCKLMANKTRILVTSKMEHLKKADKILILHEGSSYFYGTFSELQNL--QP-DFSSKLMGC---------  2 ABCC7_02    26.4%  --------------------------------GGNAILENISFSISPGQRVGLLGRTGSGKSTLLSAFLRLLN-TEGEIQIDGVSWDSITLQQWRKAFGVIPQKVFIFSGTFRKNLD-----PYEQWSDQEIWKVADEVGLRSVIEQFPGKLDFVLVDGGCVLSHGHKQLMCLARSVLSKAKILLLDEPSAHLDPVTYQII-RRTLKQAFADCTVILCEHRIEAMLECQQFLVIEENKVRQYDSIQKLLNE--RS-LFRQAISP----------  3 Sav1866     26.5%  --------------------------------NEAPILKDINLSIEKGETVAFVGMSGGGKSTLINLIPRFYDVTSGQILIDGHNIKDFLTGSLRNQIGLVQQDNILFSDTVKENILLG----RPTATDEEVVEAAKMANAHDFIMNLPQGYDTEVGERGVKLSGGQKQRLSIARIFLNNPPILILDEATSALDLESESII-QEALDVLSKDRTTLIVAHRLSTITHADKIVVIENGHIVETGTHRELIAK--QG-AYEHLYSI----------  4 MsbA_VibCh  24.3%  --------------------------------KEKPALSHVSFSIPQGKTVALVGRSGSGKSTIANLFTRFYDVDSGSICLDGHDVRDYKLTNLRRHFALVSQNVHLFNDTIANNIAYAA---EGEYTREQIEQAARQAHAMEFIENMPQGLDTVIGENGTSLSGGQRQRVAIARALLRDAPVLILDEATSALDTESERAI-QAALDELQKNKTVLVIAHRLSTIEQADEILVVDEGEIIERGRHADLLAQ--DG-AYAQLHRIQFGE------  5 MsbA_ECOli  25.2%  --------------------------------RDVPALRNINLKIPAGKTVALVGRSGSGKSTIASLITRFYDIDEGEILMDGHDLREYTLASLRNQVALVSQNVHLFNDTVANNIAYAR---TEQYSREQIEEAARMAYAMDFINKMDNGLDTVIGENGVLLSGGQRQRIAIARALLRDSPILILDEATSALDTESERAI-QAALDELQKNRTSLVIAHRLSTIEKADEIVVVEDGVIVERGTHNDLLEH--RG-VYAQLHKMQFGQ------  6 ABCB10      25.0%  -------------------------------RPEVPIFQDFSLSIPSGSVTALVGPSGSGKSTVLSLLLRLYDPASGTISLDGHDIRQLNPVWLRSKIGTVSQEPILFSCSIAENIAYGADD-PSSVTAEEIQRVAEVANAVAFIRNFPQGFNTVVGEKGVLLSGGQKQRIAIARALLKNPKILLLDEATSALDAENEYLV-QEALDRLMDGRTVLVIAHRLSTIKNANMVAVLDQGKITEYGKHEELLSK-PNG-IYRKLMNKQSFISA----  7 TM_0288     26.9%  ---------------------------------KKPVLKDITFHIKPGQKVALVGPTGSGKTTIVNLLMRFYDVDRGQILVDGIDIRKIKRSSLRSSIGIVLQDTILFSTTVKENLKYG----NPGATDEEIKEAAKLTHSDHFIKHLPEGYETVLTDNGEDLSQGQRQLLAITRAFLANPKILILDEATSNVDTKTEKSI-QAAMWKLMEGKTSIIIAHRLNTIKNADLIIVLRDGEIVEMGKHDELIQK--RG-FYYELFTSQYGLVVEKE-  8 TM_0287     28.4%  --------------------------------NTDPVLSGVNFSVKPGSLVAVLGETGSGKSTLMNLIPRLIDPERGRVEVDELDVRTVKLKDLRGHISAVPQETVLFSGTIKENLKWG----REDATDDEIVEAAKIAQIHDFIISLPEGYDSRVERGGRNFSGGQKQRLSIARALVKKPKVLILDDCTSSVDPITEKRI-LDGLKRYTKGCTTFIITQKIPTALLADKILVLHEGKVAGFGTHKELLEH--CK-PYREIYESQFGNGVMNDA  9 McjD        21.4%  ---------------------------------DKKILNSVSLDLFTGKMYSLTGPSGSGKSTLVKIISGYYKNYFGDIYLNDISLRNISDEDLNDAIYYLTQDDYIFMDTLRFNLRLA----NYDASENEIFKVLKLANL-SVVNNEPVSLDTHLINRGNNYSGGQKQRISLARLFLRKPAIIIIDEATSALDYINESEI-LSSIRTHFPDALIINISHRINLLECSDCVYVLNEGNIVASGHFRDLMVSN---EYISGLASVTE-------- ``` |

MView 1.56, Copyright © 1997-2013 Nigel P. Brown
