# Supplementary material for: Cystic Fibrosis Transmembrane Conductance Regulator (CFTR): CLOSED AND OPEN STATE CHANNEL MODELS
Source: J Biol Chem. 2015 Jul 30;290(38):22891–906. doi: 10.1074/jbc.M115.665125 (PMC4645605; doi:10.1074/jbc.M115.665125)
Supplement: Supplemental Data [file supp_M115.665125_jbc.M115.665125-1.zip › Templates.html]

# 

|  |
| --- |
| ``` Reference sequence (1): Sav1866 Identities normalised by aligned length. Colored by: property ``` |
| ``` 1 Sav1866 100.0%  ----MIK-RYLQFVKPYKYRIFATIIVGIIKFGIPMLIPLLIKYAIDGVINNHALTTDEKVHHLTIAIGIALFIFVIVRPPIEFIRQYLAQWTSNKILYDIRKKLYNHLQALSARFYANNQVGQVISRVINDVEQTKDFILTGLMNIWLDCITIIIALSIMFFL-DVKLTLAALFIFPFYILTVYVFFGRLRKLTRERSQALAEVQGFLHERVQGISVVKSFAIEDNEAKNFDKKNTNFLTRALKHTRWNAYSFAAINTVTDIGPIIVIGVGAYLAISGSITVGTLAAFVGYLELLFGPLRRLVASFTTLTQSFASMDRVFQLI-DEDYDIKNGVGAQPIEIKQGR-IDIDHVSFQYND  2 TM_0287  19.2%  ------K-TLARYLKPYWIFAVLAPLFMVVEVICDLSQPTLLARIVDEGIARG-------DFSLVLKTGILMLIVALIGAVGGIGCTVFASYASQNFGADLRRDLFRKVLSFSISNVNRFHTSSLITRLTNDVTQLQNLVMMLLRIVVRAPLLFVGGIVMAVSI-NVKLSSVLIFLIPPIVLLFVWLTKKGNPLFRKIQESTDEVNRVVRENLLGVRVVRAFRREEYENENFRKANESLRRSIISAFSLIVFALPLFIFIVNMGMIAVLWFGGVLVRNNQMEIGSIMAYTNYLMQIMFSLMMIGNILNFIVRASASAKRVLEVL-NEKPAIEEADNALALPNVEGS-VSFENVEFRYFE  3 TM_0288  24.1%  --TATLR-RLLGYLRPHTFTLIMVFVFVTVSSILGVLSPYLIGKTIDVVFVPR----R---FDLLPRYMLILGTIYALTSLLFWLQGKIMLTLSQDVVFRLRKELFEKLQRVPVGFFDRTPHGDIISRVINDVDNINNVLGNSIIQFFSGIVTLAGAVIMMFRV-NVILSLVTLSIVPLTVLITQIVSSQTRKYFYENQRVLGQLNGIIEEDISGLTVIKLFTREEKEMEKFDRVNESLRKVGTKAQIFSGVLPPLMNMVNNLGFALISGFGGWLALKDIITVGTIATFIGYSRQFTRPLNELSNQFNMIQMALASAERIFEIL-DLEEEKDDPDAVELREVRGE--IEFKNVWFSYDK  4 ABCB10   19.7%  AGLPEAR-KLLGLAYPERRRLAAAVGFLTMSSVISMSAPFFLGKIIDVIYTNP----TVDYSDNLTRLCLGLSAVFLCGAAANAIRVYLMQTSGQRIVNRLRTSLFSSILRQEVAFFDKTRTGELINRLSSDTALLGRSVTENLSDGLRAGAQASVGISMMFFV-SPNLATFVLSVVPPVSIIAVIYGRYLRKLTKVTQDSLAQATQLAEERIGNVRTVRAFGKEMTEIEKYASKVDHVMQLARKEAFARAGFFGATGLSGNLIVLSVLYKGGLLMGSAHMTVGELSSFLMYAFWVGISIGGLSSFYSELMKGLGAGGRLWELL-EREPKLPFNEGVILNEKSFQGALEFKNVHFAYPA  5 McjD     13.6%  ----LFNYIYSLMDVRGKFLFFSMLFITSLSSIIISISPLILAKITDLLSGSL----SNFSYEYLVLLACLYMFCVISNKASVFLFMILQSSLRINMQKKMSLKYLRELYNENITNLSKNNAGYTTQSLNQASNDIYILVRNVSQNILSPVIQLISTIVVVLSTKDWFSAGVFFLYILVFVIFNTRLTGSLASLRKHSMDITLNSYSLLSDTVDNMIAAKKNNALRLISERYEDALTQENNAQKKYWLLSSKVLLLNSLLAVILFGSVFIYNILGVLNGVVSIGHFIMITSYIILLSTPVENIGALLSEIRQSMSSLAGFIQRHAENKATSPSIPFLNM-ERKLN--LSIRELSFSYSD ``` |

MView 1.56, Copyright © 1997-2013 Nigel P. Brown
