# Supplementary material for: Cystic Fibrosis Transmembrane Conductance Regulator (CFTR): CLOSED AND OPEN STATE CHANNEL MODELS
Source: J Biol Chem. 2015 Jul 30;290(38):22891–906. doi: 10.1074/jbc.M115.665125 (PMC4645605; doi:10.1074/jbc.M115.665125)
Supplement: Supplemental Data [file supp_M115.665125_jbc.M115.665125-1.zip › TMDs.html]

# 

|  |
| --- |
| ``` Reference sequence (1): ABCC7_01 Identities normalised by aligned length. Colored by: property ``` |
| ``` 1 ABCC7_01   100.0%  -------KLINALRRCF----FWRFMFYGIFLYLGEVTKAVQPLLLGRIIASYDP------DNKEER-----------SIAIYLGIGLCLLFIVRTLLLHPAIFGLHHIGMQMRIAMFSLIYKKTLKLSSRVLDKISIGQLVSLLSNNLNKFDEGLALA--HFVWIAPLQVALLMGLIWEL-LQASAFCGLGFLIVLALFQAGLGRMMMKYRDQRAGKISERLVITSEMIENIQSVKAYCWEEAMEKMIENLRQTELKLTRKAAYVRYFNSSAFFFSGFFVVFLSVLPYALIK----GIILRKIFTTISFCIVLRMAVTRQFPWAVQTWYDSLGAINKIQDFLQKQEYK------TLEY---------------------NLTTTEVVMENVTAFWEE  2 ABCC7_02     7.9%  --WNTYLRYITVHKSL-----IFVLIWCLVIFLAEVAASLVVLWLLGNTPLQD-K------GNSTHSRNNSYAVIITSTSSYYVFYIYVGVADTLLAMGFFRGLPLVHTLITVSKILHHKMLHSVLQAPMSTLNTLKAGGILNRFSKDIAILDDLLPLTIF-DFIQLLLIVIGAIAVVAVL-QPYIFVATVPVIVAFIMLRAYFLQTSQQLKQLESEGRSPIFTHLVTSLKGLWTLRAFGRQPYFETLFHKALNLHTANWFLYLSTLRWFQMRIEMIFVIFFIAVTFI-SILT---TGEGEGRVGIILTLAMNIMSTL-QWAVNSSIDVDSLMRSVSRVFKFIDMPTE-GKPTKSTKPYKNGQLSKVMIIENSHVKKDDIWPSGGQMTVKDLTAKYTE  3 Sav1866     12.9%  ----MIKRYLQFVKPY-----KYRIFATIIVGIIKFGIPMLIPLLIKYAIDGVINNHALTTDEKVH------------HLTIAIGIALFIFVIVRPPIEFIRQYLAQWTSNKILYDIRKKLYNHLQALSARFYANNQVGQVISRVINDVEQTKDFILTGLM-NIWLDCITIIIALSIMFFL-DVKLTLAALFIFPFYILTVYVFFGRLRKLTRERSQALAEVQGFLHERVQGISVVKSFAIEDNEAKNFDKKNTNFLTRALKHTRWNAYSFAAINTVTDIGPIIVIGVGAYL-AISGSITVGTLAAFVGYLELLFGPL-RRLVASFTTLTQSFASMDRVFQLIDEDYDIKNGVG-AQPI---------------------EIKQGRIDIDHVSFQYND  4 TM_0288     12.8%  --TATLRRLLGYLRPH-----TFTLIMVFVFVTVSSILGVLSPYLIGKTIDVVFV------PRRF-------------DLLPRYMLILGTIYALTSLLFWLQGKIMLTLSQDVVFRLRKELFEKLQRVPVGFFDRTPHGDIISRVINDVDNINNVLGNSII-QFFSGIVTLAGAVIMMFRV-NVILSLVTLSIVPLTVLITQIVSSQTRKYFYENQRVLGQLNGIIEEDISGLTVIKLFTREEKEMEKFDRVNESLRKVGTKAQIFSGVLPPLMNMVNNLGFALISGFGGWL-ALKDIITVGTIATFIGYSRQFTRPL-NELSNQFNMIQMALASAERIFEILDLEEEKDDPD--AVEL---------------------REVRGEIEFKNVWFSYDK  5 MsbA_VibCh  11.4%  --WQTFKRLWTYIRLY-----KAGLVVSTIALVINAAADTYMISLLKPLLDEGFG------NAES-------------NFLRILPFMILGLMFVRGLSGFASSYCLSWVSGNVVMQMRRRLFNHFMHMPVRFFDQESTGGLLSRITYDSEQVAGATSRALV-SIVREGASIIGLLTLMFWN-SWQLSLVLIVVAPVVAFAISFVSKRFRKISRNMQTAMGHVTSSAEQMLKGHKVVLSYGGQEVERKRFDKVSNSMRQQTMKLVSAQSIADPVIQMIASLALFAVLFLASVD-SIRAELTPGTFTVVFSAMFGLMRPL-KALTSVTSEFQRGMAACQTLFGLMDLETERDNGKY-EA-----------------------ERVNGEVDVKDVTFTYQG  6 MsbA_EColi  10.5%  --WQTFRRLWPTIAPF-----KAGLIVAGVALILNAASDTFMLSLLKPLLDDGFG------KTDR-------------SVLVWMPLVVIGLMILRGITSYVSSYCISWVSGKVVMTMRRRLFGHMMGMPVSFFDKQSTGTLLSRITYDSEQVASSSSGALI-TVVREGASIIGLFIMMFYY-SWQLSIILIVLAPIVSIAIRVVSKRFRNISKNMQNTMGQVTTSAEQMLKGHKEVLIFGGQEVETKRFDKVSNRMRLQGMKMVSASSISDPIIQLIASLALAFVLYAASFP-SVMDSLTAGTITVVFSSMIALMRPL-KSLTNVNAQFQRGMAACQTLFTILDSEQEKDEGKR-VI-----------------------ERATGDVEFRNVTFTYPG  7 TM_0287     12.1%  ------KTLARYLKPY-----WIFAVLAPLFMVVEVICDLSQPTLLARIVDEGIA------RGDF-------------SLVLKTGILMLIVALIGAVGGIGCTVFASYASQNFGADLRRDLFRKVLSFSISNVNRFHTSSLITRLTNDVTQLQNLVMMLLR-IVVRAPLLFVGGIVMAVSI-NVKLSSVLIFLIPPIVLLFVWLTKKGNPLFRKIQESTDEVNRVVRENLLGVRVVRAFRREEYENENFRKANESLRRSIISAFSLIVFALPLFIFIVNMGMIAVLWFGGVL-VRNNQMEIGSIMAYTNYLMQIMFSL-MMIGNILNFIVRASASAKRVLEVLNEKPAIEEADN-ALAL---------------------PNVEGSVSFENVEFRYFE  8 ABCB10      14.8%  AGLPEARKLLGLAYPE-----RRRLAAAVGFLTMSSVISMSAPFFLGKIIDVIYT------NPTVD----------YSDNLTRLCLGLSAVFLCGAAANAIRVYLMQTSGQRIVNRLRTSLFSSILRQEVAFFDKTRTGELINRLSSDTALLGRSVTENLS-DGLRAGAQASVGISMMFFV-SPNLATFVLSVVPPVSIIAVIYGRYLRKLTKVTQDSLAQATQLAEERIGNVRTVRAFGKEMTEIEKYASKVDHVMQLARKEAFARAGFFGATGLSGNLIVLSVLYKGGLL-MGSAHMTVGELSSFLMYAFWVGISI-GGLSSFYSELMKGLGAGGRLWELLEREPKLPFNEGVILNE---------------------KSFQGALEFKNVHFAYPA  9 McjD        10.0%  --------LFNYIYSLMDVRGKFLFFSMLFITSLSSIIISISPLILAKITDLLSG----------------SLSNFSYEYLVLLACLYMFCVISNKASVFLFMILQSSLRINMQKKMSLKYLRELYNENITNLSKNNAGYTTQSLNQASNDIYILVRNVSQ-NILSPVIQLISTIVVVLSTKDWFSAGVFFLYILVFVIFNTRLTGSLASLRKHSMDITLNSYSLLSDTVDNMIAAKKNNALRLISERYEDALTQENNAQKKYWLLSSKVLLLNSLLAVILFGSVFIYNILG-VLNGVVSIGHFIMITSYIILLSTPV-ENIGALLSEIRQSMSSLAGFIQRHAE----NKATSPSIPF-------------------LNMERKLNLSIRELSFSYSD ``` |

MView 1.56, Copyright © 1997-2013 Nigel P. Brown
